# Genetic Analysis of the Cytoplasmic Dynein Subunit Families

**DOI:** 10.1371/journal.pgen.0020001

**Published:** 2006-01-27

**Authors:** K. Kevin Pfister, Paresh R Shah, Holger Hummerich, Andreas Russ, James Cotton, Azlina Ahmad Annuar, Stephen M King, Elizabeth M. C Fisher

## Abstract

Cytoplasmic dyneins, the principal microtubule minus-end-directed motor proteins of the cell, are involved in many essential cellular processes. The major form of this enzyme is a complex of at least six protein subunits, and in mammals all but one of the subunits are encoded by at least two genes. Here we review current knowledge concerning the subunits, their interactions, and their functional roles as derived from biochemical and genetic analyses. We also carried out extensive database searches to look for new genes and to clarify anomalies in the databases. Our analysis documents evolutionary relationships among the dynein subunits of mammals and other model organisms, and sheds new light on the role of this diverse group of proteins, highlighting the existence of two cytoplasmic dynein complexes with distinct cellular roles.

## Introduction

Dyneins are large multi-subunit protein complexes that undertake a wide range of roles within the cell. They are adenosine triphosphate (ATP)–driven, microtubule minus-end-directed molecular motors that can be divided, based on function, into two classes: axonemal and cytoplasmic dyneins [1–7] (reviewed in [8,9]). Axonemal dyneins are responsible for the movement of cilia and flagella. Two cytoplasmic dynein complexes have been identified. The most abundant cytoplasmic dynein complex, cytoplasmic dynein 1, is involved in functions as diverse as spindle-pole organization and nuclear migration during mitosis, the positioning and functioning of the endoplasmic reticulum, the Golgi apparatus, and the nucleus, and also the minus-end-directed transport of vesicles, including endosomes and lysosomes, along microtubules and retrograde axonal transport in neurons. A second cytoplasmic dynein complex, cytoplasmic dynein 2, has a role in intraflagellar transport (IFT), a process required for ciliary/flagellar assembly (reviewed in [10]).

**Figure 1:**
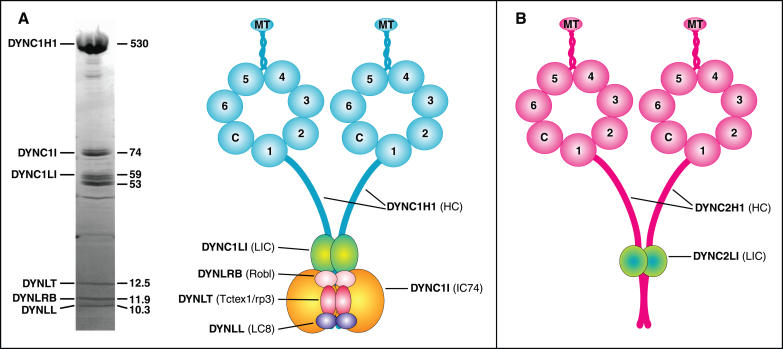
The Mammalian Cytoplasmic Dynein Complexes (A) Cytoplasmic dynein. (Left panel) Polypeptides of immunoaffinity-purified rat brain cytoplasmic dynein. Polypeptide mass (in kDa) is indicated on the right side of the gel, and the consensus family names are indicated on the left. (Right panel) Structural model for the association of the cytoplasmic dynein complex subunits. The core of the cytoplasmic dynein complex is made of two DYNC1H1 heavy chains which homodimerize via regions in their N-termini. The motor domains are at the C-termini of the heavy chains, the large globular heads of ~350 kDa that are composed of a ring of seven densities surrounding a central cavity; six of the densities are AAA domains (numbered 1–6). AAA domain 1 is the site of ATP hydrolysis. The microtubule-binding domain is a projection found on the opposite side of the ring between AAA domains 4 and 5. C is the C-terminus of the heavy chain that would form the 7th density. Two DYNC1I intermediate chains (IC74) and DYNC1LI light intermediate chains bind at overlapping regions of the N-terminus of the heavy chain, overlapping with the heavy chain dimerization domains. Dimers of the three light chain families; DYNLT, the Tctex1 light chains; DYNLRB, the Roadblock light chains; and DYNLL, the LC8 light chains, bind to the intermediate chain dimers. (B) Cytoplasmic dynein 2 complex, structural model for subunit association. This dynein complex has a unique role in IFT and is sometimes known as IFT dynein. Structural predictions indicate that the heavy chain, DYNC2H1, is similar to the cytoplasmic and axonemal dyneins. The only known subunit of this complex is a 33- to 47-kDa polypeptide, DYNC2LI1, which is related to the cytoplasmic dynein light intermediate chains. No intermediate chain or light chains have yet been identified [16].

The core of the cytoplasmic dynein 1 complex is a homodimer of two heavy chain polypeptides and associated intermediate, light intermediate, and light chain polypeptides, which are defined and named by their molecular mass and mobility in SDS-PAGE gels (Figure 1A). The protein subunits are encoded by families of at least two genes, and the expression patterns of the individual family members are different in various cell types. At least one of the light chains, DYNLL1 (LC8), has multiple cellular roles independent of its participation in a dynein complex. Cytoplasmic dynein 1 interacts with various other proteins including a second multimer, dynactin, to form the dynein–dynactin complex. Dynactin is comprised of at least seven different proteins, which together act as an adaptor that connects the cytoplasmic dynein motor to a range of cargoes (for review, see [11]). Interaction with dynactin also increases dynein motor processivity [12]. Furthermore, dynactin functions independently of dynein, anchoring microtubules at the centrosome [13]. Current evidence suggests that the second cytoplasmic dynein complex, cytoplasmic dynein 2, is also a homodimer of a distinct heavy chain, DYNC2H1, with associated light intermediate chain, DYNC2LI1 (Figure 1B). No other subunits have yet been identified for this complex, and it does not appear to interact with the dynactin complex [14–16].

The cytoplasmic dynein proteins are fundamental to the functioning of all cells, and have recently been shown to be causally mutated in forms of neurodegeneration [17–19]. They are thus of great interest for mammalian genetic, and other, studies. We therefore sought to examine the role of cytoplasmic dynein subunits from a genetic perspective. During this analysis, we noted considerable confusion in the human and mouse gene and protein names and mapping positions. Therefore, we reexamined the mapping locations for the subunit genes and clarified and updated entries in the various sequence databases. In doing so, we utilized the revised consensus nomenclature developed for the cytoplasmic dynein subunits and their genes (Table 1). We also defined, as far as possible with current data, homologous genes in model organisms, including *Drosophila, Caenorhabditis elegans, Chlamydomonas,* and yeast. To further our understanding of the function of cytoplasmic dynein subunits, we also briefly examined mutations in this group of proteins in a variety of model organisms. We do not discuss dynein-binding proteins such as dynactin, LIS1, or various kinases, which while important for dynein function, have not yet been shown to be stoichiometric components of the cytoplasmic dynein complex.

**Table 1:**
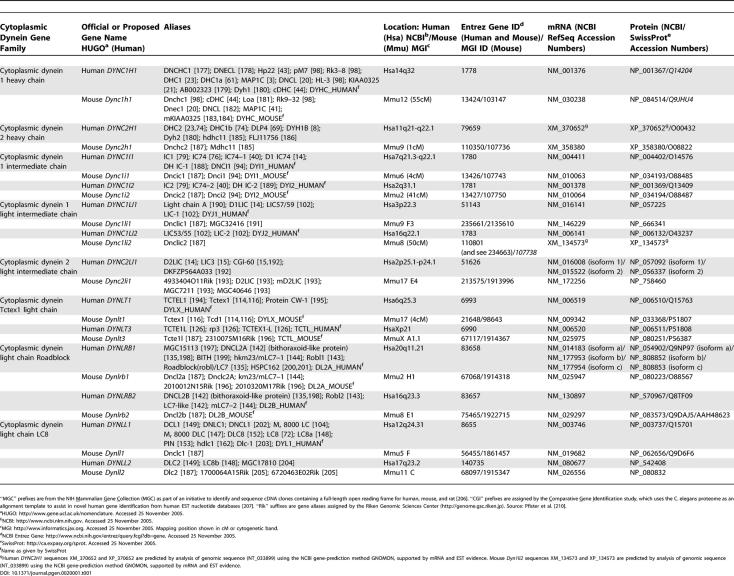
Human and Mouse Cytoplasmic Dynein Genes and Map Positions

## Human and mouse cytoplasmic dynein subunit genes.

The subunits of the cytoplasmic dynein complexes are resolved into subunit polypeptides of ~530 kDa (heavy chains), ~74 kDa (intermediate chains), ~33–59 kDa (light intermediate chains), and ~10–14 kDa (light chains) in SDS-PAGE gels (Figure 1A). Research on the cytoplasmic dynein subunits has been undertaken in a wide range of organisms from yeast to humans. The nomenclature of the mammalian genes encoding these proteins has drawn on homologs in other organisms and, consequently, a number of aliases have been found for any given human or mouse cytoplasmic dynein subunit. Much of the early research into dynein genetics was conducted in the biflagellate green alga *Chlamydomonas* on the dyneins found in the flagellar axoneme, and therefore some cytoplasmic dynein nomenclature derives from these studies. For example, mammalian members of the cytoplasmic light chain families DYNLRB and DYNLL have commonly been referred to as LC7 and LC8, respectively, which are the names of homologous *Chlamydomonas* axonemal dynein subunits.

## Nomenclature.

The revised classification system for mammalian cytoplasmic dynein (Table 1) recognizes the two distinct dynein complexes, cytoplasmic dynein 1 and cytoplasmic dynein 2, and the fact that cytoplasmic dynein light chains are shared with some axonemal dyneins. Cytoplasmic dynein subunits are also classified into polypeptide families according to sequence similarity within groups of similarly sized proteins; thus there is sequence similarity within the dynein gene families (and when cytoplasmic and axonemal members of the same gene families are compared) but not among them.

This nomenclature has been approved by the Human Genome Organization Nomenclature Committee [20] and the International Committee on Standardized Nomenclature for Mice. In accordance with their policy, the designation of each unique cytoplasmic dynein subunit starts with **DYNC** for dynein, cytoplasmic, followed by the specific dynein complex subtype **1** or **2**; for example, cytoplasmic dynein 2 is designated **DYNC2**. The shared light chains start with **DYN**. Each subunit is designated with a letter(s) for the size of the polypeptides, **H** for the heavy chain, **I** for the intermediate chain, **LI** for the light intermediate chain, and **L** for the light chain. Additional letters (**T**,** RB,** and** L**) are used to distinguish the three distinct light chain families as described in the text. Individual members of the gene families are assigned numbers. Standard human and mouse gene nomenclature is used: italicized upper case for human gene symbols (for example, *DYNC1H1*), italicized initial upper case and then lower-case letters for mouse *(Dync1h1),* and for proteins of both species, the same symbols in upper case, upright (DYNC1H1). In accordance with the International Union of Pure and Applied Chemistry standards, isoforms of the intermediate chain gene products are referred to with letters. This nomenclature system can be expanded to other subunits as appropriate. We refer to mapping positions using the prefixes Hsa *(Homo sapiens)* for human and Mmu *(Mus musculus)* for mouse, followed by the chromosomal localization e.g. Hsa2q11, Mmu11.

Table 1 lists the aliases, map position, and protein/DNA-sequence accession data for each known mouse and human cytoplasmic dynein gene. The greatest number of aliases was observed for the cytoplasmic dynein 1 heavy chain 1 *(DYNC1H1)* for which we identified 15 different names. Some alternative cytoplasmic dynein gene names have come from large-scale gene and transcript identification efforts such as the partial *DYNC1H1* clone KIAA0325 and its mouse homolog “mKIA00325,” generated by the Kazusa cDNA project [21]. A small number of gene names have been derived from the names of DNA markers and cDNA clones used to identify the genes, for example, cytoplasmic dynein 2 light intermediate chain 1, *DYNC2LI1,* was named DKFZp564A033 after the cDNA sequence and clone of the same name. The heavy chain gene *DYNC1H1* has also been referred to by the name of a marker, Hp22, generated from its human cDNA sequence, as well as the rat-derived marker Rk3–8 and a cDNA clone named HL-3.

## Cytoplasmic Dynein Heavy Chain Gene Family *(DYNC1H1, DYNC2H1)*

**Figure 2:**
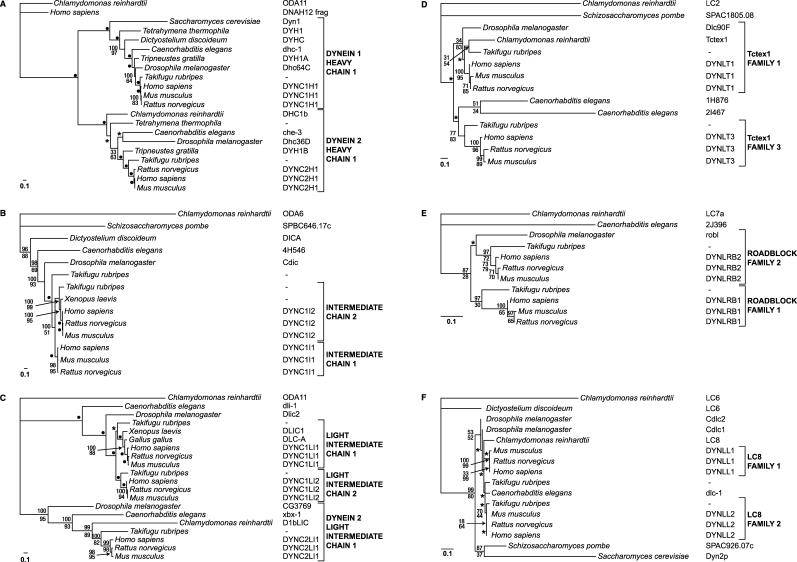
Panel Showing the Protein-Based Phylogenies of the Cytoplasmic Dynein Subunit Families Species names are shown with NCBI/GenBank gene/protein names. NCBI/GenBank protein-sequence accession numbers are given in Table S1. Orthologous human, mouse, and rat gene names use the revised systematized consensus nomenclature (e.g. DYNC1H1 in humans, mouse, and rat). Relationships amongst dynein sequences of different species do *not* necessarily reflect the evolutionary relationships amongst species; see [208] and [209] for further details. Named clades are indicated in the right margins. Bayesian and maximum-likelihood bootstrap values are shown as percentages (top and bottom, respectively), adjacent to branch points. Asterisks denote bootstraps below 50%. Filled circles denote bootstraps at 100%. Scale-bar represents evolutionary distance (estimated numbers of amino-acid substitutions per site). (A) Cytoplasmic dynein heavy chain family. *Chlamydomonas* outer arm heavy chain *(ODA11)* is used as the outgroup. DNAH12frag is the partial axonemal heavy chain fragment taken from [23]. For mouse DYNC2H1, XP_35830, only partial protein sequence (336aa) was available in the GenBank database. Adding this partial sequence to our analysis resulted in spurious clustering, therefore we obtained an extended, putative sequence by using BLAST (TBLASTN) against the mouse genome (Build 32) with human and rat sequences XP_370652 and NP_075413, respectively. Incomplete mouse genomic assembly at the *DYNC2H1* locus yielded a truncated sequence 3455 amino acids in length, 85% the length of human DYNC2H1. (B) Cytoplasmic dynein intermediate chain family. The *Chlamydomonas* IC2 *(ODA6)* is used as the outgroup. (C) Cytoplasmic dynein light intermediate chain family. There does not appear to be a sufficiently distant homolog in *Chlamydomonas* to be used as an outgroup in this analysis, therefore ODA11 (Q39610, a heavy chain protein) was chosen as the outgroup for this tree. (D) Cytoplasmic dynein light chain Tctex1 family. The *Chlamydomonas* LC2 light chain is used as the outgroup. (E) Cytoplasmic dynein light chain Roadblock family. The *Chlamydomonas* outer arm dynein LC7a, is used as the outgroup. (F) Cytoplasmic dynein light chain LC8 family. The *Chlamydomonas* Q39579 sequence is used as an outgroup. This phylogeny is poorly resolved, with low bootstrap support values and posterior clade probabilities, most likely due to there being little variation amongst the ingroup sequences. We found good support for the LC8 light chain 1 clade, and some support for the LC8 light chain 2 clade, of four vertebrate sequences. The relationships of the two sequences, C. elegans and *Takifugu* were poorly resolved, and therefore we have not included these in the LC8 light chain 2 clade.

Figure 2A shows the phylogenetic relationships amongst the dynein heavy chain protein sequences from various organisms. The heavy chain sequences fall into two distinct clades, and the relationships within each clade are generally consistent with known evolutionary distances between the organisms shown. We note that our phylogeny fits well with and extends previous phylogenetic analyses of the heavy chain proteins [22,23]. This analysis indicates that the partial human sequence DNAH12, (AAB09729) [23], is unlikely to be a cytoplasmic dynein.

## Cytoplasmic dynein heavy chain 1, DYNC1H1.

DYNC1H1, cytoplasmic dynein 1 heavy chain 1, is the largest cytoplasmic dynein subunit, having ~4,600 residues and a molecular weight of >530 kDa. First identified in rat spinal cord and brain and termed Microtubule Associated Protein 1C (MAP1C) [24], DYNC1H1 is a distant member of the AAA family of ATPases and is the cytoplasmic counterpart to axonemal dynein heavy chains [3,25]. DYNC1H1 associates as a homodimer within the cytoplasmic dynein complex and effects the contact and translocation of the dynein complex along microtubules via its large motor domain [8,26] (Figure 1B).

The C-terminal region of DYNC1H1 is the motor domain of the dynein complex and is conserved in all cytoplasmic and axonemal dynein heavy chains. This region is arranged as a heptameric ring with six AAA domains and a seventh domain, the identity of which remains a matter of discussion (Figure 1B) [12,25,27,28]. AAA domains are regions of ATP binding and hydrolysis, and thus they generate the energy required for translocation [29–31]. While the first AAA domain is essential for motor activity [32], reviewed in [30], the first four AAA domains are potentially capable of binding and hydrolysing ATP [33–35]. Contact of the heavy chain with a microtubule is established via an ~15-nm projection that extends between the fourth and fifth AAA domains [28,36]. The N-terminal region of DYNC1H1 is known as the stem, and force production, and therefore translocation, is thought to be achieved through the contact and shift of a 10-nm fold of the stem closest to the first AAA domain [37]. DYNC1H1 dimerization also occurs in the stem, and the intermediate chains and light intermediate chains bind in this region as well [38,39]. The three light chains bind to the intermediate chains [40]. Collectively the five smaller dynein subunits that bind to the N-terminus of DYNC1H1 make up the cargo-binding portion of the dynein complex.

The sequence of full-length mammalian DYNC1H1 was first obtained in rat and mouse [41,42]. Human *DYNC1H1* was identified by screening an adenocarcinoma library with a partial human cDNA [23,43]. As yet, the only mutations reported in mammalian heavy chains have been in the mouse: the *Loa* and *Cra1* strains have allelic point mutations in *Dync1h1* that cause late-onset motor neuron degeneration in heterozygotes and neuronal apoptosis in homozygotes [17]. The loss of both copies of *Dync1h1* has been shown to be lethal during early embryonic development, with disorganization of the Golgi complex, improper distribution of endosomes and lysosomes, and defects in cell proliferation; no phenotype has yet been reported for heterozygote knock-out mice [44].

In *Drosophila,* the dynein heavy chain gene, *Dhc64C,* functions in oogenesis [45,46], oocyte differentiation [47], centrosome attachment during mitosis [48], eye development, cell development in thorax, abdomen, and wing [45], and axonal transport [49]. Homozygous mutations induced by the mutagen ethyl methane sulfonate in *Dhc64C* are larval/pupal lethal, whilst heterozygotes have defects in bristle formation, eye development, and fertility [45]. In *C. elegans,* dynein heavy chain *(dhc-1)* is an essential gene, also known as *let-354* (LEThal) [50]. Extensive mutational analysis has been conducted on *dhc-1* to produce a range of variants from recessive/dominant lethals to temperature-sensitive mutants. The resultant phenotypes invariably include embryonic lethality, spindle orientation defects, polar body abnormalities, and excessive blebbing in the early embryo [51–54].

In the yeast *Saccharomyces cerevisiae,* heavy chain function ensures the alignment and orientation of mitotic spindles. Mutation of the S. cerevisiae heavy chain gene *dyn1,* which has 50% similarity (28% identity) to DYNC1H1 over 80% of the protein's length, has been shown to disrupt spindle orientation and reduce the fidelity of nuclear segregation during mitosis [55,56]. Despite this phenotype, *dyn1* mutants remain viable, although *dyn1* and kinesin double mutants are lethal [57]. This observation suggests some functional redundancy for dynein by kinesin motors in yeast. No cytoplasmic dynein 1 heavy chain 1 homolog has unambiguously been identified in *Chlamydomonas,* and neither have dyneins been found in either the Arabidopsis or rice genomes [58,59] (reviewed in [60]). There are many dynein heavy chains in the *Chlamydomonas* genome. However, with the exception of *DYNC2H1,* they appear to be components of the axonemal dyneins.

## Cytoplasmic dynein 2 heavy chain 1, DYNC2H1.

The cytoplasmic dynein 2 heavy chain, DYNC2H1, was originally identified in sea urchin embryos by Gibbons and colleagues and was termed DYH1b [61]. It is much less abundant than DYNC1H1 and does not appear to heterodimerize with DYNC1H1; biochemical analyses suggest that DYNC2H1 is a homodimer [16]. DYNC2H1 contains regions characteristic of cytoplasmic dyneins, for example, human DYNC1H1 and DYNC2H1 sequences are similar within both the motor region and around the light intermediate chain–binding site [15]. However, the expression of its mRNA increases during embryonic reciliation, a property typical of axonemal dyneins, suggesting a flagellar role for an otherwise cytoplasmic-like dynein heavy chain. The flagellar properties of DYNC2H1 were clarified with its identification as the motor responsible for retrograde (tip to base) IFT, in *Chlamydomonas,* a process required for assembly and maintenance of the eukaryotic cilium/flagellum [6,22]. DYNC2H1 is also important in modified ciliary structures such as nematode mechanosensory neurons [62] and vertebrate photoreceptors [63,64]. In *C. elegans,* the DYNC2H1 homolog, che-3, is expressed in ciliated sensory neurons which are thought to be involved in odorant chemotaxis [65]. Mutations of che-3 affect IFT, the establishment and maintenance of sensory cilia, which are stunted and swollen in the mutants, [62,66], chemotactic behavior [67], and formation of the third larval stage, dauer formation [68].

The first mammalian DYNC2H1 gene was described in rat, designated DLP4 [69], and full-length sequence has been obtained [15]. Genetic and biochemical studies suggest that DYNC2H1 associates with a member of the light intermediate chain family, DYNC2LI1 (Figure 1B, and see discussion of the light intermediate chain family below) [14,16,70–73] and possibly also with DYNLL1 (LC8) light chain [72]. In mice *Dync2h1,* mRNA is abundant in the olfactory epithelium and the ependymal layer of the neural tube; antibodies against DYNC2H1 and DYNC2LI1 strongly stain these tissues and connecting cilia in the retina as well as primary cilia of non-neuronal cultured cells [15]. The co-localization of DYNC2H1, DYNC2LI1, and homologs of the IFT pathway in mammalian ciliated tissues supports a specific role for DYNC2H1 in the generation and maintenance of mammalian cilia [14,16]. Other antibody studies suggest that DYNC2H1 localizes to the cytoplasm of apical regions of ciliated rat tracheal epithelial cells, but not in the cilia themselves [74]. In non-ciliated human COS cells, antibodies against DYNC2H1 show Golgi localization and induce Golgi dispersion, suggesting a cytoplasmic role for DYNC2H1 [23].

## Cytoplasmic Dynein Intermediate Chain Gene Family *(DYNC1I1, DYNC1I2)*

Intermediate chains are present in axonemal and/or cytoplasmic dyneins from yeast to mammals (Figure 2B). Protein-sequence data demonstrate evolutionary distant relationships between axonemal and cytoplasmic dynein intermediate chains; for example, rat DYNC1I1 has 48% similarity to the *Chlamydomonas* IC2 axonemal outer arm dynein intermediate chain encoded by the ODA6 locus [75,76]. Figure 2B shows the dynein intermediate chain protein phylogeny. The intermediate chain sequences fall into two distinct clades, intermediate chains 1 and 2, comprised of vertebrate species only. An alternative placement of a *Takifugu* sequence, as a member of the intermediate chain 1 clade, is almost as well supported by the data as the placement shown in Figure 2B (49% bootstrap support against 51% support). In view of this and with all non-vertebrate species falling outside these clades, the data suggest a recent evolutionary origin for the split into intermediate chain gene 1 and intermediate chain gene 2, perhaps as part of a “2R” event of genome duplication (see [77] for review). The absence of an amphibian *(Xenopus)* intermediate chain 1 protein may be due to the current paucity of X. laevis sequences in the GenBank sequence database (http://www.ncbi.nlm.nih.gov/Genbank).

The cytoplasmic dynein 1 intermediate chains have a molecular weight of ~74 kDa [5] and associate in the cytoplasmic dynein complex with a stoichiometry of two intermediate chains per complex [40,78]. DYNC1I1 and DYNC1I2 proteins are thought to help assemble the cytoplasmic dynein complex and to bind various cargoes. The intermediate chains interact with the dynein activator, dynactin, via their conserved N-termini [79]. The DYNC1I C-termini contain a WD repeat domain [76,80,81] that is conserved between cytoplasmic and axonemal intermediate chains and is important for intermediate chain–binding to the heavy chains [76,82]. The dynein light chains, DYNLL1 (LC8) and DYNLT1 (Tctex1), bind near the N-termini of the intermediate chains [83–85], and the DYNLRB (Roadblock) light chains bind just upstream of the WD repeat region [40]. The DYNC1I are phosphorylated, and phosphorylation at one site regulates DYNC1I2 interaction with the p150 subunit of dynactin [86,87].

The *Chlamydomonas* IC2 axonemal intermediate chain was localized to the base of the dynein heavy chain dimer by immunoelectron microscopy [88]. Steffen and colleagues identified a similar location for the cytoplasmic dynein intermediate chain and found that antibodies to it block dynein binding to membrane-bound organelles [89,90]. These data indicate a role for DYNC1I in targeting the dynein complex to various cargoes, including membranous organelles and kinetochores [76,79,89]. In *Drosophila,* mutations in dynein intermediate chain, Cdic (also referred to as cDic and Dic), lead to larval lethality, demonstrating that this intermediate chain provides an essential function. Cdic mutations dominantly enhance the rough-eye phenotype of *Glued,* a dominant mutation in the p150 subunit of dynactin [91]. *Shortwing (sw)* is an allele of the dynein intermediate chain gene but, unlike other Cdic alleles, *sw* is homozygous viable and gives rise to a recessive, temperature-sensitive defect in eye and wing development [91].

We note that in *Drosophila,* the Cdic gene lies in the 19DE region of the X chromosome, adjacent to several dynein intermediate chain-like sequences. These sequences are derived from a 7-kb duplication/deletion event involving Cdic and its proximal gene annexin X, which encodes a cell-surface-adhesion protein [92]. The duplication/deletion of this 7-kb region resulted in the formation of a de novo coding sequence, under the control of a testes-specific promoter, called sperm-specific dynein intermediate chain gene *(Sdic)* [93]. The de novo region has undergone at least 10-fold tandem duplication, which has given rise to a multi-gene family comprising at least four classes of *Sdic* gene, of which more than one class is functional [93].

### Cytoplasmic dynein 1 intermediate chain 1, DYNC1I1.

Multiple DYNC1I1 isoforms exist in mammals. They are the products of alternative splicing of the N-terminal region of a single *DYNC1I1* gene and phosphorylation [76,79,86]. In humans, alternate splicing may arise from cryptic splice-acceptor sites located within exon 4 of this 17-exon gene [94]. Two DYNC1I1 isoforms were found in rat brain and DYNC1I1 mRNA, and protein isoform expression is regulated during rat brain development, and a single DYNC1I1 isoform is found in testis. DYNC1I1 expression is also cell-specific: cultured rat neurons express at least two DYNC1I1 alternative splicing variants and their phosphorylated isoforms, while cultured glial astrocytes do not express any *DYNC1I1* gene products [95–97]. In the mouse, expression of *Dync1i1* has been shown to be restricted primarily to the brain, with weak expression in testis [94], further supporting possible neuronal specificity for *Dync1i1.* As with the other dynein subunits, the isoform diversity of the intermediate chain is thought to result in specific populations of dynein molecules that have specific functions; for example, both DYNC1I1 isoforms are components of cytoplasmic dynein found in the slow component of axonal transport in the optic nerves [95]. Multiple isoforms of the *Drosophila* intermediate chains are also produced by alternative splicing of the single gene [92].

### Cytoplasmic dynein 1 intermediate chain 2, DYNC1I2.

Vaughan and Vallee used a partial human cDNA sequence with identity to the already known *DYNC1I1* gene as a probe to isolate a rat *Dync1i2* cDNA; predicted human and rat *DYNC1I2* sequences are 94% identical [79], and the existence of two genes was supported by mapping data that placed *Dync1i1* and *Dync1i2* at distinct loci within the mouse genome [98]. Like *Dync1i1, Dync1i2* produces different splice isoforms: alternative splice sites lie at two positions within the N-terminal region. The expression of *Dync1i2* isoforms is ubiquitous with the rat DYNC1I2C isoform being expressed in all tissues and cells examined [79,94,96,97]. During rat brain development, DYNC1I2C is the only isoform found before E14 (embryonic day 14) and it is often the only isoform observed in cultured cells [96,99]. During nerve growth-factor stimulation of PC12 cell differentiation and neurite extension, there is a change in relative expression levels of the DYNC1I2 isoforms [100]. In the rat optic nerve, it has been shown that the DYNC1I2C isoform is the only intermediate chain involved in the fast component of anterograde transport to the axon tip [95,99].

The strong expression of *Dync1i2* in the mouse developing limb bud led to the suggestion that DYNC1I2 may play a role in limb development and digit patterning and/or in establishing cell polarity [94]; dynein may not do this directly, but may mediate these processes by orientating intracellular components correctly [101].

## Cytoplasmic Dynein Light Intermediate Chain Gene Family *(DYNC1LI1, DYNC1LI2, DYNC2LI1)*

Figure 2C shows the phylogenetic relationships amongst the dynein light intermediate chain protein sequences from various organisms. The light intermediate chains can be separated into three distinct groups: the two light intermediate chains that are components of cytoplasmic dynein 1, DYNC1LI1 and DYNC1LI2, are more closely related to each other than to the cytoplasmic dynein 2 light intermediate chain, DYNC2LI1. Hughes and colleagues first proposed the name light intermediate chains for these subunits [102], although these polypeptides were also referred to as light chains [103] prior to the discovery of the smaller light chains [104]. The mammalian cytoplasmic dynein complex contains four species with molecular masses of 50–60 kDa that resolve into numerous isoforms on 2D gels [86,102]. The multiple isoforms observed in 1D and 2D gels are thought to be the result of post-translational phosphorylation, although the possibility of alternate splicing has not been eliminated [86,102,103]. A third gene, *DYNC2LI1,* has recently been described which encodes a protein that appears to exclusively associate with DYNC2H1 in the cytoplasmic dynein 2 complex [14–16,71]. Unlike the other subunits of cytoplasmic dynein, homologs of the DYNC1LIs have not yet been identified in the axonemal dyneins [105]. The function of the DYNC1LIs has yet to be determined, although it has been suggested that they may regulate the interactions of dynein with dynactin, or with sub-cellular cargoes of dynein-mediated motility. DYNC1LI1 and DYNC1LI2 form only homo-oligomers, and their mutually exclusive binding to the N-terminal base of the dynein heavy chain is consistent with a role in cargo binding [38].

C. elegans appears to have one light intermediate chain *(dli-1)* for cytoplasmic dynein (DYNC1H1-based complexes), and one *(xbx-1)* for cytoplasmic dynein 2 (DYNC2H1-based complexes) [73]. *dli*-1 is required for dynein function during mitosis, pronuclear migration, centrosome separation, and centrosome association with the male pronuclear envelope [106], as well as retrograde axonal transport. Mutations in dli-1 lead to an accumulation of cargo at axonal terminals [52]. Disruption of *xbx-1* results in ciliary defects and causes behavioral abnormalities that are observed in other cilia mutants [14]. Binding of dli-1 to ZYG-12 is thought to be the mechanism for dynein binding to the nuclear envelope [107].

### Cytoplasmic dynein 1 light intermediate chain 1, DYNC1LI1.

DYNC1LI1 was cloned from rat [38] and found to have a P-loop motif, which is one of the major conserved motifs making up the nucleotide-binding domain found in numerous proteins, including ATPases and kinases [108]. DYNC1LI1, however, lacks other essential motifs associated with ATPase activity, which itself has not been assayed. Tynan showed that pericentrin, a known dynein cargo, binds DYNC1LI1 and not DYNC1LI2 [38]. DYNC1LI and its phospho-isoform are exclusively found with dynein in the slow component of axonal transport in rat optic nerves [95]. In HeLa cells, DYNC1LI1 localizes to the microtubule organizing centre and mitotic spindle, co-localizing with the GTPase Rab4a (which interacts with the central domain of DYNC1LI1 [109]); thus DYNC1LI1 may be implicated in the regulation of membrane-receptor recycling. Phosphorylation of the *Xenopus* DYNC1LI has been implicated in regulation of dynein binding to membrane-bound organelles [110]. It is thought that *Xenopus* melanosomes contain a distinct dynein light intermediate chain protein, possibly a version of DYNC1LI1 [111]. In the chicken *(Gallus gallus)* DYNC1LI1 has been called DLC-A, as part of the DLC-A group of light chains [103].

### Cytoplasmic dynein 1 light intermediate chain 2, DYNC1LI2.

DYNC1LI2 is paralogous to DYNC1LI1 and is also thought to be post-translationally modified by phosphorylation [86,102,103]. DYNC1LI2 is found in both the fast and slow components of axonal transport in rat optic nerves, although its phospho-isoforms are found only in the slow component of axonal transport. During nerve growth-factor stimulation of PC12 cell differentiation and neurite extension, *DYNC1LI2* gene expression is up-regulated [112], and phosphorylation of both DYNC1LI1 and DYNC1LI2 is increased [100]. Like DYNC1LI1, the chicken *(G. gallus)* DYNC1LI2 has also been termed DLC-A, as part of the DLC-A group of light chains [103].

### Cytoplasmic dynein 2 light intermediate chain 1, DYNC2LI1.

*DYNC2LI1* is a light intermediate chain that was identified in mammals by two groups and was originally designated D2LIC [14] and LIC3 [15]. DYNC2LI1 is the light intermediate chain that associates with DYNC2H1 in the cytoplasmic dynein 2 complex: Grissom and colleagues observed that DYNC2LI1 co-immunoprecipitated specifically with DYNC2H1 and co-localized with DYNC2H1 at the Golgi apparatus. Mikami and coworkers [15] found the 350-amino acid LIC3 polypeptide (AAD34055) had a 24% similarity to rat DYNC1LI2 but failed to observe Golgi localization. DYNC2LI1 has been identified in mouse, *C. elegans, Drosophila,* and *Chlamydomonas* [14,16]. A targeted deletion of *Dync2li1* in mouse affects development, in particular ventral cell fates and axis establishment in the early embryo [113]. In *Chlamydomonas,* DYNC2LI1 (D1bLIC) is essential for retrograde IFT [71]. As mentioned above, DYNC2LI1 appears to bind exclusively with DYNC2H1; in agreement with this, we find DYNC2LI1 homologs in species that have DYNC2H1. The exclusive association of DYNC2H1 and DYNC2LI1 with one another, and not with any of the other cytoplasmic dynein subunits, emphasizes the distinct cellular identities and roles of these separate DYNC1H1 and DYNC2H1 dynein complexes.

## Cytoplasmic Dynein Light Chain Gene Families

There are three known dynein light chain gene families that are components of cytoplasmic dynein 1: (1) the *t*-complex–associated family *(DYNLT1, DYNLT3),* (2) the Roadblock family *(DYNLRB1, DYNLRB2),* and (3) the LC8 family *(DYNLL1, DYNLL2).* The gene families are named according to their original discovery, through the effect of mutations in mouse (*t*-complex associated, Tctex1) and *Drosophila* (Roadblock), or according to the size of the protein in *Chlamydomonas* (LC8) as discussed below. We present each family by molecular weight, starting with the largest light chain protein gene family, the *t*-complex–associated family (~113 amino acids), through to the Roadblock family (~96 amino acids) and the smallest light chain proteins, the LC8 family (~89 amino acids). As described below, some of the light chains have cellular functions that are independent of their role in the cytoplasmic dynein 1 complex.

## Cytoplasmic Dynein Light Chain Tctex1 Gene Family *(DYNLT1, DYNLT3)*

Figure 2D shows the phylogenetic relationships amongst the dynein light chain Tctex1-family protein sequences from various organisms. Our phylogeny shows distinct clades for DYNLT1-like and DYNLT3-like sequences. Tctex2-like sequences lie closer to the outgroup than they do to the DYNLT1 and DYNLT3 clades (not shown).

### Cytoplasmic dynein light chain Tctex1, DYNLT1.

*Tctex1* (*t-c*omplex *t*estis-*ex*pressed) gene was originally identified within the mouse *t-complex* (a 30- to 40-Mb region of Mmu17) as a candidate for one of the “distorter” products responsible for the non-Mendelian transmission of variant *t* haplotypes [114]. Lader et al. [114] and O'Neill and Artzt [115] found evidence of four copies of *Dynlt1 (Tctex1)* in the mouse genome; we found that the current genomic sequence databases appear to contain only one such locus that maps to Mmu 17, although a processed pseudogene has also been described on Mmu6. Subsequently, DYNLT1 was found to be an integral component of cytoplasmic dynein [116], and has since also been identified within axonemal inner and outer arm dyneins [117,118]. DYNLT1 binds to the N-terminus of the intermediate chain DYNC1I [85]. Many studies have identified DYNLT1 as a binding partner for various cellular proteins, and it has been suggested that it may attach specific proteins or cellular components to cytoplasmic dynein; for example, DYNLT1, but not its homolog DYNLT3 (see below), binds to the C-terminal domain of rhodopsin and is required for the trafficking of this visual pigment within photoreceptors [119]. The two DYNLT1 polypeptides in the cytoplasmic dynein complex dimerize, and their dimer structure is similar to that of the DYNLL1, LC8, dimer [116,120–122]. The evidence suggests that the same *Dynlt1* gene product is a component of both axonemal and cytoplasmic dyneins in mouse [117]. The binding site on DYNC1I for DYNLT1 has been mapped to a 19-amino acid region at the N-terminus [85].

The Schizosaccharomyces pombe DYNLT-like gene SPAC1805.08 (also referred to as Dlc1) is involved in movement of nuclear material during meiotic prophase and is expressed in astral microtubules and microtubule-anchoring sites on the cell cortex. The Dlc1 localization pattern is similar to that of cytoplasmic dynein heavy chain Dhc1 [123]. Dlc1 null mutants are viable but have irregular nuclear movement during meiosis and defects in sporulation, recombination, and karyogamy [123]. Genetic analyses in *Drosophila,* which appears to have only one member of the DYNLT family, suggest that DYNLT1 is not essential for cytoplasmic dynein function, as the null mutation is not lethal. However, the mutants do have sperm-motility defects, suggesting they do have an essential role in axonemal dynein [124,125]. In *Chlamydomonas,* Tctex1 is an axonemal inner arm dynein component [117], and recently a variant form has been identified in axonemal outer arm dynein (DiBella et al., in press).

### Cytoplasmic dynein light chain, DYNLT3.

Closely related to *DYNLT1* is *DYNLT3,* also known as rp3 because it was initially a candidate for causing X-linked retinitis pigmentosa type 3 [126]. However, the actual gene that is defective in this disease was later identified as a guanine nucleotide exchange factor that is unrelated to *DYNLT3* [127]. Subsequently, King and colleagues found that DYNLT3 is a cytoplasmic dynein light chain that is differentially expressed in a cell- and tissue-specific manner [78,116]. Interestingly, while many proteins have been identified as binding partners for DYNLT1, none have been identified as binding exclusively to DYNLT3, though recently the Herpes simplex virus capsid protein VP26 has been shown to bind both DYNLT1 and DYNLT3 [128]. There is no evidence that DYNLT3 is a component of axonemal dyneins.

### Axonemal dynein light chain, *Tctex2.*

To avoid confusion with Tctex2, an axonemal dynein subunit, the DYNLT2 designation is not used: a third human *t*-*c*omplex *t*estis-*ex*pressed gene, originally characterized by Rappold and colleagues [129,130], was given the name *Tctex2,* and is also known as LC2, *TCTE3,* and *Tcd3.* Patel-King and colleagues demonstrated that it has 35% identity to the 19,000-M_r_ (relative mobility) axonemal outer arm dynein light chain (LC2) of *Chlamydomonas* [131], and that it is distantly related to cytoplasmic light chains DYNLT1 and DYNLT3 [116,120]. LC2 is essential for outer arm dynein assembly [132]. There is evidence that Tctex2 may interact substoichiometrically with cytoplasmic dynein, but there has not yet been a definitive demonstration that it is a cytoplasmic dynein subunit.

In mice, expression of *Tctex2* is testis-specific, particularly in later spermatogenic stages, and isoforms are thought to be generated by alternative splicing [130]. As yet, isoforms of the human homolog have not been identified, and its expression is restricted to tissues containing cilia and flagella [133]. Mutations in *Tctex2* have been implicated in the autosomal recessive disorder primary ciliary dyskinesia, which results in the impairment of ciliary and flagellar function, although these mutations are thought not to be the primary cause of the disorder [133].

Mouse *Tctex2* lies within the Mmu17 *t*-complex in a central region containing the distorter/sterility locus *Tcd3* [134]. Human *Tctex2* maps to the long arm of Chromosome 6 [129] and, interestingly, is a neighbor of the two genes, *TCP1* and *TCP10,* which are also homologs of mouse *t*-complex loci found adjacent to mouse *Tctex2.* This conservation of gene order suggests that the region of Chromosome 6q containing these genes is syntenic to the homologous central region of mouse Chromosome 17. In contrast, DYNLT1 and DYNLT3 are located on human Chromosome 6p and show synteny to the distal portion of the mouse *t*-complex, suggesting that the middle and distal portions of the mouse *t*-complex are syntenic to the long and short arms of human Chromosome 6, respectively [129].

## Cytoplasmic Dynein Light Chain Roadblock Gene Family *(DYNLRB1, DYNLRB2)*

The first Roadblock gene was identified in *Drosophila* through mutational analyses*,* and from biochemical and sequence comparisons with the *Chlamydomonas* outer arm dynein LC7a light chain [135,136]. *Drosophila* has at least six Roadblock homologs, including bithoraxoid, which has been implicated in thoracic and abdominal parasegment development. These proteins belong to an ancient family that has been implicated in NTPase regulation in bacteria [137]. Mutations in the Roadblock genes result in the accumulation of axonal cargoes, mitotic defects, female sterility, and either larval or pupal lethality [135]. Roadblock mutations also affect neuroblast proliferation and result in reduced dendritic complexity, as well as in defects in axonal transport [138]. Mutational analysis in *Chlamydomonas* suggests that DYNLRB (LC7a) is involved in axonemal outer arm dynein assembly, and a related protein (LC7b) is associated with dynein regulatory elements [136,139].

Figure 2E shows the phylogenetic relationships amongst the dynein light chain Roadblock-family protein sequences from various organisms. The Roadblock sequences are remarkably well conserved between different organisms, with 96% of pair-wise sequence comparisons amongst all sequences shown in Figure 2E demonstrating an identity greater than 50% (data not shown). The high conservation of Roadblock-family sequences presumably arises from functional constraints on the proteins. We note that genes in mammals and in other species incorporate conserved and complete Roadblock sequences (known as Roadblock domains) within their coding regions [135,137]. However, these genes are not thought to be cytoplasmic dyneins; for example, MAPBPIP in human and mouse appears to function mainly in the endosome/lysosome pathway [140]. Both DYNLRB polypeptides are found in mammalian cytoplasmic dynein, but it is not yet known if just one, or both, are utilized in mammalian axonemal dyneins.

## Cytoplasmic dynein light chain Roadblock1, DYNLRB1.

Database searches [135,141] (Figure 2E) revealed there are two Roadblock-related proteins in mammals, DYNLRB1 and DYNLRB2 (also termed DYNLC2A and DYNLC2B) [142]. Biochemical studies suggest that in mammals both Roadblocks exist as homo- and heterodimers that associate with cytoplasmic dynein [143] through specific binding sites on the intermediate chains, distinct from those for the DYNLL (LC8) and DYNLT (Tctex1) light chains [40]. Expression studies in humans have identified tissue-specific differences in the expression of the two human Roadblock-like genes, with strong expression of *DYNLRB1* in heart, liver, and brain, and up-regulation in hepatocellular carcinoma tissues [142]. In a role that may be independent of its association with cytoplasmic dynein, TGFb phosphorylation of human DYNLRB1 (termed mLC7–1/km23 by Tang and colleagues [144]) results in the human DYNLRB1 binding to the TGFb receptor that mediates TGFb responses including JNK activation, c-JUN phosphorylation, and growth inhibition.

### Cytoplasmic dynein light chain Roadblock 2, DYNLRB2.

*DYNLRB2* was identified by EST database searches for sequences homologous to *Chlamydomonas* LC7a [135,141]; human *DYNLRB2* was cloned in 2001 [142] and was found to be differentially expressed in various tissues, including hepatocellular carcinomas.

## Cytoplasmic Dynein Light Chain LC8 Gene Family *(DYNLL1, DYNLL2)*

### Cytoplasmic dynein light chain LC8 1, DYNLL1.

DYNLL (the light chain that has been known as LC8, as well as LC8a and PIN) is a component of many enzyme systems, and it has a long and somewhat confusing history. This protein was originally identified, using biochemical methods, as a light chain of the *Chlamydomonas* axonemal outer arm dynein [145,146]. The term LC8 derives from the observation that this component migrates at ~8 kDa in SDS-PAGE gels, and it is also the smallest of the eight light chains then known within this *Chlamydomonas* axonemal dynein. It was first cloned from *Chlamydomonas,* and closely related sequences were identified in mouse and nematode along with more distantly related proteins in higher plants [147,148]. Using biochemical and immunochemical methods, DYNLL was also identified as an integral component of brain cytoplasmic dynein [104]. Only recently, it has been realized that mammals have two closely related DYNLL genes, and that the protein products of both genes are components of cytoplasmic dynein [148,149]. Thus, most of the studies on the cellular roles of DYNLL do not distinguish between the two DYNLL polypeptides.

Another factor complicating efforts to elucidate the role of the DYNLL polypeptides in dynein function was the realization that large amounts of DYNLL1 in brain, and presumably cells in general, are not associated with the dynein complex [104]. In fact, the DYNLL polypeptides have other important functions unrelated to their role in axonemal and cytoplasmic dyneins. DYNLL1 is a subunit of the flagellar radial spokes which are involved in control of axonemal dynein motor function [150]. DYNLL1 is also a substrate of a p21-activating kinase, and its interaction with the kinase may be important for cell survival [151]. A DYNLL is an integral component of the actin-based motor myosin V [152]. Immunostaining shows that a DYNLL is concentrated in dendritic spines and growth cones, and it is proposed that this is due to its association with the actin-based motor myosin V [149]. DYNLL1 was identified within neuronal nitric oxide synthase (nNOS) [14] and named “PIN” for “protein inhibitor of nNOS” [153]. However, it is unclear whether it is actually an inhibitor of nNOS or is merely a component of the nNOS complex, as DYNLL1 appears to be required for the stability of various multimeric enzyme complexes. DYNLL1 has been found to interact with a wide variety of other cytoplasmic components, including the pro-apoptotic factor Bim [154], *Drosophila* swallow [155,156], and rabies virus P protein [157], and it may act to attach them to the dynein and/or myosin-V molecular motors. In addition, there are *many* other DYNLL-interacting proteins not mentioned here that have been identified using yeast two-hybrid screens and other methods.

There are two copies of DYNLL in the cytoplasmic dynein complex, and the crystal and NMR structures of the DYNLL dimer with bound peptide are known [104,158–160]. Both monomers contribute to the formation of two symmetrical grooves in the dimer that are the binding sites for two DYNC1I polypeptides reviewed in [161]. Adding DYNLL to an N-terminal polypeptide of DYNC1I in vitro increases the structural order of DYNC1I, suggesting that DYNLL is important for the assembly of a functional dynein complex [84].

Figure 2F shows the phylogenetic relationships amongst the dynein light chain LC8-family protein sequences from various organisms. Our phylogeny shows that the mammalian LC8 light chain family falls into two distinct clades containing DYNLL1- and DYNLL2-like genes. DYNLL is highly conserved from alga and humans, and homologs are required for sensory axon projection and other developmental events in *Drosophila* [162,163], nuclear migration in *Aspergillus* [164], and retrograde IFT in *Chlamydomonas* [72]. The phenotype of partial loss-of-function mutants in *Drosophila* revealed a wide array of pleiomorphic developmental defects; the total loss-of-function mutation was embryonic lethal [162]. The *Drosophila* dynein light chain 1 (Cdlc1, also known as ddlc1 and “cut-up” *[ctp]*) is ubiquitously expressed during development and in adult tissue, and is required for proper embryogenesis and cellular differentiation. Mutations in this gene result in female sterility, which may be due to the severely disordered cytoskeletons of ovarian and embryonic cells [162]. A high degree of sequence similarity (92%) exists between *Drosophila* Cdlc1 and the 8-kDa flagellar outer arm dynein light chain from *Chlamydomonas,* and with human and C. elegans light chain 1 (91%), suggesting this gene has been under strong selective pressure [162]. S. pombe has a single known DYNLL homolog, SPAC926.07c (also referred to as Dlc2); it is transcribed during the vegetative phase, induced at low level in the sexual phase, and is enriched at the nuclear periphery [123]. A Dlc2 null mutant has been described with marginally reduced recombination in meiosis, but no other reported phenotype [123].

During the course of homology searches for this paper, we noted that *DYNLL1* has related sequences in several locations in the human genome (data not shown); none of these appear to be associated with expressed sequences and thus may be pseudogenes. There was also a discrepancy in the likely mapping position of *DYNLL1,* and therefore we carried out a sequence analysis of *DYNLL1*-related genomic loci and show that the cognate human locus lies on Hsa12q24.31 (data not shown), which agrees with the mouse mapping result of *Dynll1* on Mmu5.

### Cytoplasmic dynein light chain LC8 2, DYNLL2.

DYNLL2, also known as DYNLL2 and LC8b, is the second member of this light chain family. It was identified by micro-sequencing of polypeptides from purified brain cytoplasmic dynein [148] and a yeast two-hybrid screen [149]. Mammalian DYNLL1 and DYNLL2 have 93% identity, differing by only six amino acids out of 89. Indicative of the extraordinary conservation of these proteins, the amino acid sequences of both DYNLL1 and DYNLL2 from human, mouse, rat, pig, and cow are identical [148]. Human DYNLL2 was identified in a yeast two-hybrid screen using the guanylate kinase–associated protein (GKAP) as bait and may mediate the interaction between GKAP and actin- and microtubule-based motors, allowing GKAP and its associated proteins to be translocated as a cargo, although DYNLL1 also binds to GKAP [149]. DYNLL2 binds the pro-apoptotic factor Bmf, which binds Bcl2, neutralizing its antiapoptotic activity, a role comparable to that reported for the binding of Bim to DYNLL1 [165]. However, it has also been observed that Bim and Bmf have identical binding affinities for both DYNLL1 and DYNLL2 [166].

It has further been proposed that DYNLL1 binds specifically to the dynein intermediate chain DYNC1I, while DYNLL2 binds to the myosin-V heavy chain. However, DYNLL2 co-purifies with cytoplasmic dynein from various rat tissues [148], and DYNLL1- and DYNLL2-GST are equally effective in binding myosin V [149]. Furthermore, DYNLL1 and DYNLL2 bind with equal affinity to DYNC1I in pair-wise yeast two-hybrid studies (K. W. Lo and K. K. Pfister, unpublished data). It is not yet known if one, or both, of the DYNLL polypeptides are associated with axonemal dyneins; however, DYNLL1 is enriched in testes and lung—tissues that have large numbers of cilia or flagella [148].

## Human and Mouse Cytoplasmic Dyneins: Nomenclature, Map Positions, and Sequences

To create Table 1, we cataloged, by literature searches, all known gene and protein names for the cytoplasmic dyneins in mouse and human. In addition, aliases were recorded from the single-query interface LocusLink (http://www.ncbi.nlm.nih.gov/LocusLink) and the Mouse Genome Informatics (MGI) website (http://www.informatics.jax.org). We also included aliases previously approved by the HUGO Gene Nomenclature Committee (http://www.gene.ucl.ac.uk/nomenclature) as well as aliases referenced in sequence submissions to the GenBank (http://www.ncbi.nlm.nih.gov/Genbank) and Entrez (http://www.ncbi.nlm.nih.gov/entrez) sequence databases [167].

Human and mouse orthologs in Table 1 are taken from the literature and databases. Human and mouse chromosomal locations were obtained from the literature and from the MGI and LocusLink databases. The OMIM numbers given for gene and disease loci in humans refers to the unique accession numbers in the On-line Mendelian Inheritance in Man database (http://www.ncbi.nlm.nih.gov/entrez/query.fcgi?db=OMIM). Nucleotide and protein sequences (prefix NM_ and NP_, respectively) are National Center for Biotechnology Information (NCBI) Reference Sequence (RefSeq, http://www.ncbi.nlm.nih.gov/RefSeq) and Swiss-Prot accession numbers (http://www.ebi.ac.uk/swissprot), respectively [167]. The NCBI RefSeq project provides a non-redundant and comprehensive collection of nucleotide and protein sequences drawn from the primary-sequence database GenBank. RefSeq collates and summarizes primary-sequence data to give a minimal tiling path for individual transcripts, using available cDNA and genomic sequence whilst removing mutations, sequencing errors, and cloning artifacts. Sequences are validated in silico by NCBI's Genome Annotation project to confirm that any genomic sequence incorporated into a RefSeq cDNA matches primary cDNA sequences in GenBank, and that the coding region really can be translated into the corresponding protein sequence. Accession numbers beginning with the prefix XM_ (mRNA) and XP_ (protein) are RefSeq sequences of transcripts and proteins that are annotated on NCBI genomic contigs; these may have incomplete cDNA-tiling-sequence data or contig sequences [168]. For dyneins with known isoforms, isoform-sequence accession numbers available within nucleotide and protein databases are given.

Included in the heavy chains that we found were “Cell Division Cycle 23, yeast homolog” *(CDC23)* and “Cell Division Cycle 22, yeast homolog” *(CDC22),* which are GenBank aliases for human and mouse dynein heavy chain 1, respectively. We found no evidence in the literature to support the “CDC” designation of these genes and their products in terms of either “Cell Division Cycle” or “Cytoplasmic Dynein Chain”. We compared mouse and human heavy chain 1 cDNA and protein sequences with mouse, human, and yeast CDC23 and CDC22 sequences and found no similarity to support this designation (data not shown). We concluded that the synonym CDC had most likely been attributed in error, and we contacted NCBI who, in agreement with our findings, removed the CDC designation from the sequences involved.

## Human/Mouse Homology Searches

Homology searches of human cytoplasmic dynein subunit genes were conducted using position-specific iterative BLAST (PSI-BLAST) [169] at NCBI (http://www.ncbi.nlm.nih.gov/BLAST; Table 2). The PSI-BLAST program identifies families of related proteins using an iterative BLAST procedure [170]. In an initial search, a position-specific scoring matrix is constructed from a multiple sequence alignment of the highest scoring hits. Subsequent iterations using the position-specific scoring matrix are performed in a new BLAST query to refine the profile and find additional related sequences. We used nucleotide and protein sequences from each known human dynein gene to query the human and mouse non-redundant sequence databases at GenBank, using default parameters and the BLOSUM-62 substitution matrix, which has been shown to be the most effective substitution matrix to identify new members of a protein family [171]. Where dynein isoforms were present, the longest sequence was used to search the databases.

## Phylogenetic Analysis

To establish gene family groupings, we investigated the phylogenetic relationships between dynein protein homologs in various organisms. Homologous sequences were identified by searching the GenBank non-redundant protein database, with the human protein using PSI-BLAST with default parameters and the BLOSUM-62 substitution matrix. Searches of pufferfish sequence Takifugu rubripes (commonly known as Fugu rubripes), for which little transcribed sequence exists although a usable genome assembly is present, were performed using the BLAST (TBLASTN) feature at the Ensembl Fugu Genome Browser (version 2.0; http://www.ensembl.org/Fugu_rubripes), searching with human protein sequence against a translated nucleotide database.

Protein sequences were aligned for comparison across their full lengths using the multiple sequence alignment program CLUSTALW [172] (http://www.ebi.ac.uk/clustalw) and applying the GONNET250 matrix as default. The GONNET250 is a widely used matrix for performing protein-sequence alignments, allowing 250 accepted point mutations per 100 amino acids, using scoring tables based on the PAM250 matrix [173].

Two different phylogenetic methods were used to analyse the dynein gene family alignments. Maximum-likelihood trees were inferred under the Jones, Taylor, and Thornton (JTT) empirical model of amino-acid substitution using PHYML version 2.4.3 [174], as was non-parametric bootstrapping using 100 resampled alignments for each gene family. Bayesian analyses were performed using MrBayes version 3.0B4 [175], using the default Bayesian priors on tree topologies and branch lengths. Two different sets of analyses were performed for each gene family, the first allowing the Markov-chain Monte-Carlo algorithm to move between the 11 different amino-acid substitution models available in MrBayes, and another specifying the JTT model. The first analysis allows the chain to take into account uncertainty in the substitution process. For all analyses performed here, the posterior probability of the JTT model was at least 99%, confirming that this model best describes the evolution of the dynein sequences—so only results from the fixed-JTT model analyses are shown here.

For each analysis, three chains of 1,000,000 generations each were run, sampling parameters every 100 generations and discarding the first 100,000 generations as a burn-in period. Running these multiple independent chains allowed visual confirmation that the chains had reached a stationary state by ensuring that all three chains were moving around a region of similar likelihood. For one of the gene families (cytoplasmic dynein heavy chain), the three chains had reached different likelihood values after 1,000,000 generations, suggesting failure to converge. Running another three independent chains resulted in five out of six chains agreeing on the likelihood values, suggesting that only one chain had not converged properly. In all cases, the phylogeny presented is the majority-rule consensus of the posterior sample of tree topologies from all three Markov chains, drawn using TreeView [176] with posterior clade probabilities and maximum-likelihood bootstrap values shown for each clade on these trees.

## Searching for Function and Mutant Phenotypes

As well as literature searches, information on protein function was taken from the Gene Ontology database (http://www.geneontology.org), which provides data on function and processes associated with a search protein. Mutant-phenotype data were obtained from the literature and the following sources: Online Mendelian Inheritance in Man at NCBI for human (http://www.ncbi.nlm.nih.gov/entrez/query.fcgi?db=OMIM); MGI for mouse (http://www.informatics.jax.org); FlyBase for *Drosophila* (http://www.flybase.org); and WormBase for C. elegans (http://www.wormbase.org).

## Conclusions

In this paper, we have provided an overview of the two cytoplasmic dynein complexes, cytoplasmic dynein 1 and cytoplasmic dynein 2, from a genetic perspective. We have highlighted the unique subunit compositions and cellular functions of the two cytoplasmic dyneins, and we have emphasized the unique role of cytoplasmic dynein 2 in IFT. We have described the different mammalian dynein gene families, and have shown the phylogenetic and functional relationships between members of individual families. We carried out initial database searches and clarified and corrected anomalous data. We have also discussed known functions and mutations of these proteins, and we have highlighted both their fundamental importance to the cell and the fact that much research remains to be carried out to define the roles of individual proteins.

## Supporting Information

Table S1 Species Names, NCBI/GenBank Protein-Sequence Accession Numbers, and NCBI/GenBank Gene/Protein Names for Figures 2A–F (53 KB DOC)

### Accession Numbers

The Entrez Gene database (http://www.ncbi.nlm.nih.gov/entrez) accession numbers for the proteins discussed in this paper are Cdic (also referred to as cDic and Dic) (44160); che-3 (DYNC2H1 homolog) (172593); DYNC1I1 (1780); DYNC1I2 (1781); DYNC1LI1 (51143); DYNC1LI2, (1783); DYNC2H1, (79659); DYNC2LI1 (51626); DYNLL1 (LC8) (8655); DYNLL2 (also known as DYNLL2 and LC8b) (140735); DYNLRB1 (also termed DYNLC2A) (83658); DYNLRB2 (also termed DYNLC2B) (83657); DYNLT1 (Tctex1) (6993); MAPBPIP (28956); SPAC1805.08 (also referred to as Dlc1) (3361491).

The Entrez Gene database (http://www.ncbi.nlm.nih.gov/entrez) accession numbers for the genes discussed in this paper are *Dhc64C* (38580); *dli-1* (178260); *dyn1* (853928); *Dync1h1* (13424); *DYNC1H1* (1778) **;** *Dync1i1* (13426); *Dync1i2* (13427); *Dync2li1* (213575); *Dynlt1* (*Tctex1*) in the mouse genome (21648); *DYNLT3* (6990); human *Tctex2* (also known as LC2, *TCTE3,* and *Tcd3*) (6991); mouse *Tctex2* (21647); *xbx-1* (184080).

The Entrez Protein database (http://www.ncbi.nlm.nih.gov/entrez/query.fcgi?db=protein&cmd=search&term=) accession numbers for the proteins discussed in this paper are C. elegans LC8 sequence (49822)*; C. elegans* light chain 1 (498422); Cdlc1 (525075); *Chlamydomonas* 19,000-M_r_ axonemal outer arm dynein light chain (LC2) (AAB58383); rat DYNC1I1 (062107); rat DYNC1LI2 (112288).

The OMIM (http://www.ncbi.nlm.nih.gov/entrez/query.fcgi?db=OMIM) accession numbers for the proteins discussed in this paper are autosomal recessive disorder primary ciliary dyskinesia (242650); Bim (603827); Bmf (606266); X-linked retinitis pigmentosa type 3 (300389).

The Ensembl (http://www.ensembl.org/Fugu_rubripes/textview) accession number for the *Takifugu* LC8 sequence is SINFRUP0000015498.

The SwissProt (http://ca.expasy.org/sprot/) accession numbers for the *Chlamydomonas* 8-kDa flagellar outer arm dynein light chain and the *Chlamydomonas* LC2 light chain used as the outgroup in Figure 2 are Q39580 and T08216, respectively.

## Abbreviations

ATP: adenosine triphosphate
BBSRC: Biotechnology and Biological Sciences Research Council
IFT: intraflagellar transport
JTT: Jones, Taylor, and Thornton
MGI: Mouse Genome Informatics
Mr: relative mobility
NCBI: National Center for Biotechnology Information
NIH: National Institutes of Health
nNOS: neuronal nitric oxide synthase
PSI-BLAST: position-specific iterative BLAST
RefSeq: Reference Sequence
