# Supplementary material for: Genetic Analysis of the Cytoplasmic Dynein Subunit Families
Source: PLoS Genet. 2006 Jan 27;2(1):e1. doi: 10.1371/journal.pgen.0020001 (PMC1331979; doi:10.1371/journal.pgen.0020001)
Supplement: Supplementary file 1 [file pgen.0020001.st001.doc]

|  | **Species name** | **Accession number** | **Name/Symbol** |
| --- | --- | --- | --- |
| **HEAVY**  **CHAIN** | *Chlamydomonas reinhardtii*  *Homo sapiens*  *Saccharomyces cerevisiae*  *Tetrahymena thermophila*  *Dictyostelium discoideum*  *Caenorhabditis elegans*  *Tripneustes gratilla*  *Drosophila melanogaster*  *Takifugu rubripes*  *Homo sapiens*  *Mus musculus*  *Rattus norvegicus*  *Chlamydomonas reinhardtii*  *Tetrahymena thermophila*  *Caenorhabditis elegans*  *Drosophila melanogaster*  *Tripneustes gratilla*  *Takifugu rubripes*  *Rattus norvegicus*  *Homo sapiens*  *Mus musculus* | Q39610  AAB09729  NP_012980  AAK30570  P34036  NP_491363  CAA79935  NP_523929  SINFRUG00000157004  NP_001367  NP_084514  NP_062099  CAB56748  AAB81841  NP_492221  NP_609857  AAA63583  SINFRUG00000149357  NP_075413  XP_370652  - | ODA11  DNAH12 frag  Dyn1  DYH1  DYHC  dhc-1  DYH1A  Dhc64C  -  DYNC1H1  DYNC1H1  DYNC1H1  DHC1b  -  che-3  Dhc36D  DYH1B  -  DYNC2H1  DYNC2H1  DYNC2H1 |
| **INTERMEDIATE**  **CHAIN** | *Chlamydomonas reinhardtii*  *Schizosaccharomyces pombe*  *Dictyostelium discoideum*  *Caenorhabditis elegans*  *Drosophila melanogaster*  *Takifugu rubripes*  *Takifugu rubripes*  *Xenopus laevis*  *Homo sapiens*  *Rattus norvegicus*  *Mus musculus*  *Homo sapiens*  *Mus musculus*  *Rattus norvegicus* | P27766  NP_595375  P54703  NP_501038  NP_477075  SINFRUP00000144032  SINFRUP00000173453  AAH68704  NP_001369  NP_446332  NP_034194  NP_004402  NP_034193  NP_062107 | ODA6  SPBC646.17c  DICA  4H546  Cdic  -  -  -  DYNC1I2  DYNC1I2  DYNC1I2  DYNC1I1  DYNC1I1  DYNC1I1 |
| **LIGHT**  **INTERMEDIATE**  **CHAIN** | *Chlamydomonas reinhardtii*  *Caenorhabditis elegans*  *Drosophila melanogaster*  *Takifugu rubripes*  *Xenopus laevis*  *Gallus gallus*  *Homo sapiens*  *Rattus norvegicus*  *Mus musculus*  *Takifugu rubripes*  *Homo sapiens*  *Rattus norvegicus*  *Mus musculus*  *Drosophila melanogaster*  *Caenorhabditis elegans*  *Chlamydomonas reinhardtii*  *Takifugu rubripes*  *Homo sapiens*  *Rattus norvegicus*  *Mus musculus* | Q39610  NP_502518  NP_727501  SINFRUP00000161076  AAG42486  Q90828  NP_057225  NP_665715  NP_666341  SINFRUP00000141300  NP_006132  NP_112288  XP_134573  NP_609289  NP_506683  AAQ12259  SINFRUP00000130818  NP_057092  XP_216632  NP_758460 | ODA11  dli-  Dlic2  DLIC1  -  DLC-A  DYNC1LI1  DYNC1LI1  DYNC1LI1  -  DYNC1LI2  DYNC1LI2  DYNC1LI2  CG3769  xbx-1  D1bLIC  -  DYNC2LI1  DYNC2LI1  DYNC2LI1 |
| **Tctex1**  **FAMILY** | *Chlamydomonas reinhardtii*  *Schizosaccharomyces pombe*  *Drosophila melanogaster*  *Chlamydomonas reinhardtii*  *Takifugu rubripes*  *Homo sapiens*  *Mus musculus*  *Rattus norvegicus*  *Caenorhabditis elegans*  *Caenorhabditis elegans*  *Takifugu rubripes*  *Homo sapiens*  *Rattus norvegicus*  *Mus musculus* | T08216  NP_593919  NP_477356  T07930  SINFRUP00000154636  NP_006510  NP_033368  NP_112608  NP_492063  NP_495718  SINFRUP00000163662  NP_006511  XP_343770  NP_080251 | LC2  SPAC1805.08  Dlc90F  Tctex1  -  DYNLT1  DYNLT1  DYNLT1  1H876  2I467  -  DYNLT3  DYNLT3  DYNLT3   |
| **ROADBLOCK**  **FAMILY** | *Chlamydomonas reinhardtii*  *Caenorhabditis elegans*  *Drosophila melanogaster*  *Takifugu rubripes*  *Homo sapiens*  *Rattus norvegicus*  *Mus musculus*  *Takifugu rubripes*  *Homo sapiens*  *Mus musculus*  *Rattus norvegicus* | AAD45881  NP_495943  NP_523771  SINFRUP00000136252  NP_570967  XP_341695  NP_083573  SINFRUP00000147978  NP_054902  NP_080223  NP_571985 | LC7a  2J396  robl  -  DYNLRB2  DYNLRB2  DYNLRB2  -  DYNLRB1  DYNLRB1  DYNLRB1 |
| **LC8**  **FAMILY** | *Chlamydomonas reinhardtii*  *Dictyostelium discoideum*  *Drosophila melanogaster*  *Drosophila melanogaster*  *Chlamydomonas reinhardtii*  *Mus musculus*  *Rattus norvegicus*  *Homo sapiens*  *Takifugu rubripes*  *Caenorhabditis elegans*  *Takifugu rubripes*  *Mus musculus*  *Rattus norvegicus*  *Homo sapiens*  *Schizosaccharomyces pombe*  *Saccharomyces cerevisiae* | Q39579  AAO51837  NP_477408  NP_525075  Q39580  NP_062656  NP_445771  NP_003737  SINFRUP00000154968  NP_498422  SINFRUP00000136749  NP_080832  NP_542428  NP_542408  NP_594368  NP_010712 | LC6  LC6  Cdlc2  Cdlc1  LC8  DYNLL1  DYNLL1  DYNLL1  -  dlc-1  *-*  DYNLL2  DYNLL2  DYNLL2  SPAC926.07c  Dyn2p   |
